# Cognitive mediated eye movements during the SDMT reveal the challenges with processing speed faced by people with MS

**DOI:** 10.1186/s12883-019-1543-8

**Published:** 2019-12-26

**Authors:** Bennis Pavisian, Viral P. Patel, Anthony Feinstein

**Affiliations:** 1 Department of Psychiatry, Sunnybrook Health Sciences Centre, 2075 Bayview Avenue, Toronto, Ontario M4N 3M5 Canada; 2 Department of Psychiatry, University of Toronto, 2075 Bayview Avenue, Toronto, Ontario M4N 3M5 Canada

**Keywords:** Multiple sclerosis, Eye movements, Eye tracking, Symbol digits modalities test, Cognition, Processing speed

## Abstract

**Background:**

The Symbol Digit Modalities Test (SDMT) is regarded as the cognitive test of choice for people with MS (pwMS). While deficits are linked to impaired processing speed, the mechanisms by which they arise are unclear. Cognitive-mediated eye movements offer one putative explanation. The objective of this study was to determine the association between eye movements and performance on the SDMT.

**Methods:**

Thirty-three people with confirmed MS and 25 matched healthy control subjects (HC) were administered the oral SDMT while eye movements were recorded.

**Results:**

Mean SDMT scores were significantly lower in pwMS (*p* < 0.038). Shorter mean saccade distance in the key area (*p* = 0.007), more visits to the key area per response (*p* = 0.014), and more total number of fixations in the test area (*p* = 0.045) differentiated pwMS from HCs. A hierarchical regression analysis revealed that the number of visits to the key area per response (*p* < 0.001; ΔR^2^ = 0.549) and total number of fixations in the test area (p < 0.001; ΔR^2^ = 0.782) were the most robust predictors of SDMT scores.

**Conclusion:**

Cognitive-mediated eye movements help elucidate the processing speed challenges confronted by people with MS. Mechanistic insights such as these can potentially help inform new cognitive rehabilitation strategies.

## Background

Cognitive dysfunction casts a wide shadow in the lives of people with MS (pwMS). The prevalence ranges from 40 to 70% [1, 2] and the negative effects are apparent when it comes to finding and sustaining employment, [3] pursuing leisure activities, [4] maintaining relationships [5] and driving a motor vehicle [6]. Detection of deficits is therefore essential, more so as recent data suggest that cognitive rehabilitation can bring about significant benefits [7].

The neuropsychological literature points towards the Symbol Digit Modalities Test (SDMT) as the single best cognitive test for pwMS. It is quick, patient friendly, easily translatable, sensitive and valid, requires minimal equipment, and carries the imprimatur of consensus agreement [8, 9]. Given the prominence now afforded with this measure, a closer look at its properties is necessary to gain a better understading of how it has emerged from an extensive neuropsychological choice to reach this point. The SDMT is considered primarily a test of information processing speed, one of the core cognitive deficits in pwMS [10]. However, it is also apparent that other factors are implicated in performance. To date two have been explored, namely oral-motor ability [11] and incidental visual memory [12]. In relation to the former, slowed speech secondary to neurological factors may hinder performance on a test in which the anwers, given verbally, are speed dependent. As to the latter, results on the SDMT have been linked not only to information processing speed, but also to incidental memory that can contribute to approximately 10% of the performance variability. In addition, a third factor, namely eye movement, may be potentially relevant to performance on the SDMT [10]. While there is a substantial literature devoted to eye movement abnormalities in general in pwMS, [13–16] and links established with cognitive dysfunction, [17, 18] reported associations with impairments on the SDMT are equivocal [19–21]. Of note is that none of the three SDMT studies monitored eye movements during the actual performance of the task, rather, assocations or a lack thereof were reported between SDMT performance on the one hand, and separate measurements of eye movement, on the other. Allied to this observation is another, namely that the majority of MS-SDMT studies do not take visual abnormalities into account in their methodology and interpreation of results [10]. Given this relative dearth of information, it is informative to look beyond the MS literature where associations between eye-tracking metrics and performance on the SDMT have been found in people with schizophrenia [22] and Huntington’s disease [23].

In light of the above uncertainties allied with the need to gain a more complete understanding of the mechanisms underlying SDMT impairment, we have undertaken a study in which the aim is to explore the relationship between eye movements and performance on the standard, oral version of the SDMT.

## Methods

### Participants

Subjects included 33 people with a confirmed diagnosis of MS (pwMS) and 25 healthy controls (HC) matched for age, sex, and years of education. All participants were between the ages of 18 and 60 years. Exclusion criteria included no prior traumatic brain injury, a diagnosis of another central nervous system condition, a relapse during the past month, substance abuse, psychosis, intellectual handicap, and previous neuropsychological testing within the past year and no corticosteroid use in the past month. Visual acuity was assessed with a Snellen Chart and only participants with normal or corrected to normal vision were included. Eye spectacles were not a contraindication for participation in the study as this did not prevent eye tracker calibration.

### Data collection

Demographic and neurologic data included age, gender, years of education, marital status, EDSS, disease duration and course. Neurology case notes were examined for details of abnormal findings with respect to cranial nerves II, III, IV and VI that could affect eye movements. In some cases there was gap in time (which did not exceed 3 months) between the neurological examination and the eye-tracking examination. Premorbid IQ was assessed with the Weschler Test of Adult Reading (WTAR) [24]. Symptoms of depression and anxiety were measured using the Hospital Anxiety and Depression Scale (HADS) [25, 26].

### Eye tracking

The eye tracking data were collected using the Gazepoint GP3 HD eye tracker [27] attached to a desktop computer. The GP3 HD recorded eye movement data at a sampling rate of 150 Hz with 0.5–1.0 degree of visual angle accuracy. Binocular data were streamed using the Gazepoint API. The data were recorded and analyzed binocularly on the OGAMA (Open Gaze and Mouse Analyzer) software [28]. The eye tracking metrics chosen were those that directly indicated evidence of neurologic dysfunction, i.e. saccade velocity and others that were markers of cognitively mediated eye movements such as time and number of fixations in designated areas of interest.

The SDMT was displayed in its original paper form [29] and the exact spatial dimensions of the test were reproduced on a 19-in. flat panel monitor with a standard resolution of 1280 × 1024. Thus, the size of the symbols and numbers and the key and test areas were an exact replica of the original paper version of the SDMT. As with the paper version of the SDMT, the test was presented for 90 s and responses were recorded orally.

During testing, the participant’s head was secured to a chin rest that was placed 20 in. from the computer monitor. A nine-point calibration was then performed via the Gazepoint Application Program Interface (API). Once calibration was completed, a feedback cursor of the participant’s eye movements would appear on the screen. Participants were asked to move their eyes and to fixate on the “bullseye” of targets on the screen to ensure eye movements were recorded accurately. Subjects were instructed to keep their heads as still as possible throughout the calibration and performance of the test. When calibration failed, as it did in three healthy control subjects and five pwMS, these subjects were excluded from the study. The final sample size of 33 pwMS and 25 HC represents complete, valid eye-tracking data sets.

For the purposes of eye-tracking, the SDMT was divided into two rectangular areas of interest (AOI) using the OGAMA program, namely the key and the test areas. The key area refers to the two rows of boxes located at the top of the page, one filled with numbers 1 to 9 and the other with the nine symbols linked to each number. The test area refers to the area of the page containing rows of symbols that must be matched to the correct number according to the key. Eye tracking measurements included the following: total number of fixations (defined as maintaining of the visual gaze on a single location) in the test and key area, mean fixation time in the test and key area, total number of visits to the key area, number of visits to key area per response, average number of fixations in key and test area per visit, average time spent in the key and test areas per visit, mean saccade velocity and mean saccade distance.

The eye metrics were collected for each AOI and for the total area of the test. The percentage of data out of the monitor was also recorded. With a 150 Hz sample rate, there was no loss of tracking of the patients’ eyes.

### Statistics

After confirming the normality of data distribution, between group comparisons were undertaken with independent t-tests and chi square analyses for continuous and ordinal data respectively. Predictors of performance on the SDMT were sought with a hierarchical linear regression analysis. Only those demographic and psychiatric variables that differed significantly between the MS and HC groups were considered as potential predictors of performance on the SDMT. Prior to running the hierarchical regression, correlations between the predictor variables were explored to prevent entry of two highly correlated variables. Effect sizes were calculated for each between group comparison.

### Informed consent

The current study received research ethics approval from Sunnybrook Hospital and written consent was obtained from all participants.

## Results

### Demographic, neurologic and psychometric data

There were no demographic differences between the MS and HC groups (see Table 1). All MS participants had relapsing-remitting disease. The mean EDSS score for the MS sample was 2.33 (SD = 1.84, median = 2.0 and range = 0–6.0). There were no abnormalities in cranial nerves II, III, IV and VI in individuals with MS. The MS group had significantly higher scores than the HC subjects on the HADS-depression (5.25 (3.69) vs. 2.04 (1.94), t = − 3.75, p - .001) and HADS-anxiety (8.64 (4.21) vs. 3.57 (3.44), t = − 4.64, *p* = .001) scales.

**Table 1:** Demographic, neurologic and psychiatric comparison between MS and HC groups

	MS (*n* = 33); Mean (SD), n (%)	HC (*n* = 25); mean (SD), n (%)	t-test/x^2^	*p*-value	Cohen’s d
Demographic					
Age	41.42 (9.89)	38.52 (12.73)	t = − 0.941	*p* = 0.351	0.254
Gender (% female)	22 (70%)	13 (57%)	X^2^ = 1.192	*p* = 0.397	0.143
Years of education	14.71 (2.13)	15.56 (1.12)	t = 1.750	*p* = 0.086	0.490
Premorbid IQ	104.59 (8.28)	104.06 (7.07)	t = − 0.220	*p* = 0.827	0.069
Neurologic					
EDSS, median (range)	2.00 (0–6)				
Neuropsychological testing					
SDMT	41.06 (10.37)	46.35 (6.72)	t = 2.267	*p* = 0.038	0.610
Psychiatric					
HADS – Depression	5.25 (3.69)	2.04 (1.94)	t = −3.751	*p* < 0.001	1.09
HADS - Anxiety	8.64 (4.21)	3.57 (3.44)	t = − 4.643	*p* < 0.001	1.32

Abbreviations: *MS* Multiple Sclerosis, *HC* Healthy Controls, *HADS* Hospital Anxiety and Depression Scale, *SDMT* Symbol Digit Modalities Test

### Behavioral performance on SDMT

The MS group had significantly lower SDMT mean scores than the healthy control group (41.0 (10.37) vs. 46.35 (7.07), t-2.27, *p* = .038). Seven (21.21%) MS participants were impaired on the SDMT, as defined by a score of 1.5 standard deviations below the mean performance of the healthy control subjects.

### Eye tracking

The comparison of eye tracking metrics between the MS and HC groups are found in Table 2. The MS group had significantly more fixations in the test area, more visits to the key area per response and a shorter mean saccade distance in the key area. Less than 0.94% of data were out of monitor for the MS group and 1.0% of data for the controls, which did not differ significantly (*p* = 0.937).

**Table 2:** Comparison of eye tracking metrices between MS and HC groups

	MS (*n* = 33); mean (SD), n (%)	HC (*n* = 25); mean (SD), n (%)	t-test/x^2^	*p*-value	Cohen’s d
Total number of fixations in the test area	129.26 (38.41)	110.00 (33.53)	t = −2.054	*p* = 0.045	0.534
Total number of fixations in the key area	161.13 (55.98)	163.52 (46.56)	t = 0.167	*p* = 0.868	0.046
Mean fixation time in the test area (ms)	182.97 (66.98)	182.17 (30.16)	t = −0.053	*p* = 0.958	0.015
Mean fixation time in the key area (ms)	154.29 (21.09)	160.78 (33.81)	t = 0.867	*p* = 0.390	0.230
Total number of visits to the key area	44.71 (11.69)	40.48 (8.72)	t = −1.460	*p* = 0.150	0.410
Number of visits to key area per response	1.19 (0.53)	0.88 (0.24)	t = −2.545	p = 0.014	0.753
Average number of fixations in the key area per visit	3.73 (0.80)	4.04 (1.05)	t = 1.251	*p* = 0.216	0.332
Average number of fixations in the test area per visit	3.00 (0.54)	2.82 (1.30)	t = −0.672	*p* = 0.504	0.181
Average time spent in the key area per visit (ms)	556.71 (143.17)	626.21 (119.69)	t = 1.899	*p* = 0.063	0.526
Average time spent in the test area per visit (ms)	523.96 (129.93)	460.24 (122.74)	t = −1.836	*p* = 0.072	0.504
Mean saccade velocity, total (degrees/ms)	0.066 (0.017)	0.064 (0.011)	t = −0.422	*p* = 0.649	0.128
Mean saccade distance, total (degrees)	4.73 (0.71)	4.99 (0.96)	t = 1.18	*p* = 0.265	0.288
Mean saccade distance in the key area (degrees)	2.89 (0.40)	3.36 (0.80)	t = 2.83	*p* = 0.007	0.733
Mean saccade distance in the test area (degrees)	2.73 (0.63)	2.68 (0.77)	t = −0.32	*p* = 0.751	0.086
Mean saccade velocity in the key area (degrees/ms)	0.024 (0.001)	0.026 (0.001)	t = 0.876	*p* = 0.385	0.244
Mean saccade velocity in the test area (degrees/ms)	0.027 (0.01)	0.025 (0.001)	t = −0.824	*p* = 0.414	0.229

Abbreviations: *MS* Multiple Sclerosis, *HC* Healthy Controls

### Predictors of performance on the SDMT

A correlation analysis showed that none of the eye metrics used in the regression model had a correlation higher than 0.480 with one another. One variable, HADS-Anxiety, was removed from the regression model due to a correlation coefficient of r = 0.740 with HADS-Depression. The remaining putative predictor variables entered into the hierarchical analysis were: Step 1 = group (MS vs. control subject); Step 2 = HADS depression scores: The inclusion of the HADS depression variable at Step 2 was justified not only by the highly significant between group difference but also by data from studies showing that depression can have a negative effect on processing speed [30, 31].; Step 3 = education: The inclusion of education at Step 3 was based on a MS versus HC effect size of 0.49., Step 4 = total mean saccade distance in the key area; Step 5 = number of visits to key area per response; Step 6 = number of fixations in the test area. The choice of the eye tracking metrics was confined to those three variables that differed significantly between the MS and control groups with their order of entry in the analysis determined by the degree to which they differed between groups.

The results of the hierarchal regression are shown in Table 3. The regression revealed that at step 1, Group (MS versus HC) membership was a significant predictor of SDMT performance (F = 4.540; *p* = 0.038) with 6.3% of the variance. At step 2, Group and HADS depression scores were not significant (F = 0.963; *p* = 0.331) and accounted for 6.2% of the variance. Step 3 of the model included years of education, which was also not significant (F = 0.791; *p* = 0.404). At Step 4, adding mean saccade distance did not add significantly to the variance (F = 0.850; *p* = 0.361). At step 5, the addition of number of visits to the key area per response was shown to be the strongest predictor of SDMT performance (F = 55.212; *p* < 0.001) with 54.9% of the variance. Step 6, which includes all the four variables plus the total number of fixations in the test area, was significant (F = 53.564; p < 0.001) contributing 78.2% of the variance.

**Table 3:** Hierarchal regression analysis of SDMT performance

Model	R	R^2^	ΔR^2^	Significance
1^a^	0.283	0.080	0.063	*p* = 0.038
2^b^	0.312	0.097	0.062	*p* = 0.331
3^c^	0.322	0.103	0.049	*p* = 0.404
4^d^	0.335	0.112	0.059	*p* = 0.361
5^e^	0.768	0.590	0.543	*p* < 0.001
6^f^	0.902	0.813	0.786	*p* < 0.001

^a^Group;

^b^Group*HADS-Depression;

^c^Group*HADS-Depression*Years of Education

^d^Group*HADS-Depression*Years of Education*Mean Saccade Distance in the key area

^e^Group*HADS-Depression*Years of Education*Mean Saccade Distance in the key area*Number of Visits to the Key per response;

^f^Group*HADS-Depression*Years of Education*Mean Saccade Distance in the key area * Number of Visits to the Key per response* Total number of fixations in test area

## Discussion

Our study confirmed that people with MS perform slower on the SDMT than healthy individuals and revealed that eye movement metrics are predictive of performance. Significantly, certain eye movement metrics contributed appreciably more to the variance in SDMT performance than education and depression combined. These findings are potentially significant because the SDMT has been called a ‘sentinel’ test for cognitive impairment in MS [32] with the Multiple Sclerosis Outcome Assessments Consortium (MSOAC) extolling its numerous attributes, including effectiveness in measuring processing speed deficits, strong test-retest reliability, and robust correlations with MRI measurements of atrophy, lesion burden, and pathology [9].

When interpreting the eye movement metrics in people with MS it is helpful to divide them into two broad categories. The first may arise in response to pathological changes in the relevant cranial nerves and brain stem and cerebellar circuits that directly control eye movement. These abnormalities, detectable on routine neurological examination, may include loss of visual acuity and diminutions of visual fields, different forms of strabismus, various types of nystagmus, impaired vestibular-ocular response (as typically seen in intranuclear ophthalmoplegia), and horizontal gaze paresis [14]. Such gross disturbance in ocular function could impair performance of a visual based test such as the SDMT. Of note is that in our MS group, confined to people with relapsing-remitting disease course with a median EDSS of 2.0 indicative of mild physical disability, no participant was found to have signs attributable to abnormalities in cranial nerves II, III, IV or VI which could have influenced eye movements.

In addition, more subtle abnormalities involving saccadic eye movements that are not detectable on clinical examination may also in theory impair performance on visual-based cognitive tests. Saccades too are under tight neurological control and MS may directly impair such saccadic metrics as latency, amplitude and velocity [33]. Our data revealed that one of these variables, namely mean saccadic distance was significantly reduced in the key area for the MS group when compared to healthy control subjects.

The second broad category refers to the possibility of MS subtly disrupting the cognitive control of mechanisms that oversee eye movements. Attention, working memory and decision making that is part of executive functioning can all influence the saccadic network. Indeed, a multifocal disease like MS is well placed to disrupt the synchronization of widely dispersed neural circuits that link ocular centers in frontal regions such as the frontal eye fields, supplementary frontal eye fields, dorsolateral prefrontal cortex and anterior cingulate cortex with those in the posterior parietal cortex. Dysfunctional attentional control of eye movements in response to distractors [19] and impaired attention disrupting memory-guided saccades [17] are two examples of this.

The SDMT eye tracking literature in people with MS is confined to three studies, none of which measured eye movements while subjects completed the test. In the first study, with the report limited to an abstract, a modest, albeit significant correlation was reported between performances on the SDMT and the K-D test, the latter considered indicative of saccade velocity [21]. The second, more substantive study, made use of an antisaccade (AS) paradigm, a marker of attention and executive processes that guides response selection and inhibition [20]. Twenty-four people with relapsing remitting MS and 14 healthy control subjects were administered the AS test and a brief cognitive battery and all participants underwent a brain MRI. More AS errors were found in the MS group and correlated significantly with performances on the SDMT and the Paced Auditory Serial Addition Test (PASAT), but not the California Verbal Learning Test. The imaging findings were notable for an association between AS errors and MRI indices of cerebellar pathology, including focal grey matter atrophy and global cerebellar mean diffusivity and fractional anisotropy changes. This result, however, only partly replicates an earlier AS study in which more errors were associated with impairments on the PASAT, but not the SDMT [19].

Our study adds to this nascent literature, being the first to track eye movements during the completion of the SDMT. While we showed that mean saccadic distance in the key area was significantly reduced in people with MS, this change did not independently predict performance on the SDMT. Rather, what differentiated the responses of people with MS from healthy individuals were the number of visits to the key area per response followed by an increase in the total number of fixations in the test area. What these cognitively mediated eye tracking measurements therefore clearly expose are the uncertainties and delays that characterize the performances of people with MS when given the relatively simple task of matching a number and symbol at speed. The mechanistic underpinnings to these disordered responses reflect the key role played by attention in controlling eye movements [17] with the knock on cognitive effects, as it were, manifesting as delayed information processing speed. In addition, the strong association between quicker performance on the SDMT and fewer visits to the key per response suggests that incidental visual memory is also influencing eye movements during the task.

Findings from a schizophrenia study using a similar methodology corroborate these conclusions. Compared to healthy individuals, those with schizophrenia spent significantly more time in the key area, had a higher number of fixations in the test and key areas, and had a higher number of visits to the key area for each response. As with our study, a regression analysis revealed that the number of visits to the key area per response was the most significant predictor of SDMT performance. The researchers concluded that the between-group differences found were largely explained by this variable [22]. Negative findings from a Parkinson’s disease (PD) study are, however, harder to interpret given a substantially different methodology [33]. The paper and pencil version of the SDMT requiring written responses was used and the eye-tracking device was attached to the heads of participants making it problematic to control for movement. Furthermore, sample size was small with only 12 subjects in the PD group deemed to be cognitively impaired. Finally, a study of people with Huntington’s disease (HD) showed that those with eye movement abnormalities, measured clinically before proceeding to cognitive testing, had more deficits on the SDMT and a number of other tests than people with HD who had normal eye movements [23].

Our study, while informative and providing good construct validity for the SDMT as a test of processing speed, nevertheless contains certain limitations which suggest that our conclusions should be considered preliminary. Participants did not complete a more comprehensive cognitive battery that could have added further insights into the origins of differences in task-related eye movements between people with MS and healthy individuals. In addition, our sample was small (only seven people with MS were impaired on the SDMT) and composition was homogenous containing only people with relapsing-remitting multiple sclerosis (RRMS) with mild disability. This meant that our failure to find a causative link between non-cognitively mediated saccadic metrics, for example saccadic velocity, and slowed SDMT performance, could be due to type II error. Sample limitations also prevented the exploration of a potential tripartite relationship between increasing disease burden, greater cognitive impairment and more extensive eye movement abnormalities. What is known is that cognitive impairment in people with secondary progressive forms of the disease is almost double that found in RRMS [1, 34]. Similarly, eye movement abnormalities increase with higher EDSS scores [14]. What is not known, and what our data cannot address, is whether these findings are linked.

## Conclusion

In conclusion, our study sheds new light on one of the mechanisms underlying the difficulties people with MS have in performing the Symbol Digits Modalities Test. Mechanistic understandings can in turn potentially help with the development of new cognitive rehabilitation strategies. For example, data from stroke patients show that computer-driven visual training programs tailored according to the type of cognitive deficit, can lead to improvements in attention, memory, executive function and spatial orientation [35]. As such, the insights to emerge from our MS eye movements data, while incomplete, seem worthy of further research.

## Data Availability

Subject data are stored in Dr. Anthony Feinstein’s Neuropsychiatry laboratory in Sunnybrook Research Institute. The data are available on request.

## Abbreviations

AOI: Areas of interest
API: Application Program Interface
AS: Anti-saccade
EDSS: Expanded Disability Status Scale
HADS: Hospital Anxiety and Depression Scale
HC: Healthy controls
HD: Huntington’s Disease
MSOAC: Multiple Sclerosis Outcome Assessments Consortium
OGAMA: Open Gaze and Mouse Analyzer
PASAT: Paced Auditory Serial Addition Test
PD: Parkinson’s disease
pwMS: People with Multiple Sclerosis
RRMS: Relapsing-remitting multiple sclerosis
SDMT: The Symbol Digit Modalities Test
WTAR: Weschler Test of Adult Reading
